# High dietary protein and energy content does not alter hepatic and renal health blood markers in growing lambs

**DOI:** 10.1007/s11250-026-05053-2

**Published:** 2026-05-28

**Authors:** Fernando Correia Cairo, Cláudia Loianny Souza Lima, Susiane de Carvalho Matos, Marcia Pereira Silva, Gisele Rocha dos Santos, Evandro Pereira Neto, Melquizedeque Pontes Ribas Santos, Lays Débora Silva Mariz, Elzania Sales Pereira, José Augusto Gomes Azevedo, Gleidson Giordano Pinto de Carvalho, Douglas dos Santos Pina, Stefanie Alvarenga Santos

**Affiliations:** 1 Department of Animal Science, Federal University of Bahia, Salvador, BA 40.170-110 Brazil; 2 Department of Animal Science, Federal University of Ceara, Fortaleza, CE 60.020-181 Brazil; 3 Department of Agricultural and Environmental Sciences, Santa Cruz State University, Ilhéus, BA 45.662-900 Brazil

**Keywords:** Health, Nutrition, Ruminants, Urea

## Abstract

This study aimed to evaluate the effect of different concentrate levels and increasing crude protein content in the diet on blood markers of hepatic and renal health in growing lambs. Two experiments were conducted with 64 non-castrated male Santa Inês lambs, four months old, with an initial average weight of 20 ± 1 kg. The lambs were housed in individual pens and fed ad libitum during a 57-days experimental period, with 14-days adaptation period. In Experiment 1 (Exp1), four diets with different concentrate levels (400, 500, 600, and 700 g/kg of DM) were tested, and in Experiment 2 (Exp2), four diets with different crude protein levels (120, 140, 160, and 180 g CP/kg of DM) were evaluated. Blood samples were collected on the 54^th^ day in both experiments for analysis of serum albumin, urea, cholesterol, creatinine, alkaline phosphatase (AP), alanine aminotransferase (ALT), aspartate aminotransferase (AST), and gamma-glutamyltransferase (GGT). The results indicated that the increase in digestible organic matter intake (dOM) did not affect the blood parameters analyzed (*p* > 0.05). However, serum urea was above the reference range (17–43 mg/dL) in Exp1, while creatinine was below the reference range (1.2–1.9 mg/dL). In Exp2, the increase in crude protein intake led to a linear increase in serum urea (*p* = 0.0002), although no changes were observed for AP, albumin, AST, ALT, GGT, creatinine, and cholesterol (*p* > 0.05). This study demonstrated that increasing intake of dOM and CP above the requirements in growing lambs raises serum urea without affecting liver or renal health.

## Introduction

The biochemical profile is a valuable nutritional and metabolic assessment tool in production animals (Schultz et al. 2022). Many metabolic markers can be used to evaluate nutrient adequacy in a diet. For accurate interpretation, factors such as species, breed, climate, and physiological state are essential (Onasanya et al. 2015). Dietary changes can overload the liver and kidneys, resulting in alterations in blood markers of hepatic and renal health (Zhang et al. 2017).

The metabolic profile and complete blood count are important tools for monitoring metabolic adaptation, identifying nutritional imbalances, and aiding disease prevention (Russell 1991). These tests are particularly relevant in animals subjected to more intensive production systems, such as feedlots, where increased nutritional intake promotes rapid weight gain and shortens the production cycle through diets with higher proportions of concentrate. Enzymes such as alanine aminotransferase (ALT), aspartate aminotransferase (AST), gamma-glutamyl transferase (GGT), and alkaline phosphatase (AP) are markers of hepatic injury (Xue et al. 2025), and their activity can indicate the location and degree of cellular damage (González and Silva 2022).

Studies showed that diets with high levels of protein and concentrates can increase blood levels of urea and other metabolites, indicating an additional effort by the body to metabolize these nutrients (Wang et al. 2020). However, in many cases, increased protein intake does not result in greater utilization of this protein by the body (Wang et al. 2021). Diets rich in concentrates, with a high intake of non-fibrous carbohydrates, can cause metabolic and digestive disturbances, such as acidosis, in addition to overloading the liver and kidneys (Ahmed et al. 2022).

The structure and function of the liver underscore the critical importance of eliminating ammonia, which is potentially toxic to ruminants. Enzymes involved in the ornithine cycle and transamination reactions are strategically located in the mitochondria and cytosol of periportal and perivenous hepatic cells. This arrangement allows for the conversion of ammonia, absorbed in the intestine, into urea and also enables the use of glutamine synthesis as an additional mechanism to remove almost all the ammonia present in the hepatic portal blood (Meijer et al. 1990; Katz 1992).

We hypothesized that high concentrate ratios and a crude protein content above the nutritional requirements in the diet could alter blood markers of hepatic and renal health, above and below the reference values, in growing lambs. Then, the objective of this work was to evaluate the effect of different concentrate ratios and increase dietary protein content on the blood markers of hepatic and renal health in growing lambs.

## Materials and methods

### Ethics, location of experiments and environment.

Two experiments were carried out at the Experimental Farm of the Federal University of Bahia (UFBA), located in the municipality of São Gonçalo dos Campos, Bahia State, Brazil. The farm is located in a hot tropical region with consistently high temperatures (24 to 28°C) and a marked rainy season from April to July. Annual rainfall ranges from 800 to 1,200 mm, typical of the semihumid tropical climate of coastal Bahia, at coordinates 12◦23’57.51’‘S and 38◦52’44.66’’ W. The experiments were carried out during the summer, outside the rainy season, when environmental conditions are less favorable for parasite development and infestation. The chemical analyses of the collected samples were analyzed in the Laboratory of Animal Nutrition of the same institution, located in the municipality of Salvador, Bahia State, Brazil. All procedures involving animals were conducted in accordance with the guidelines established by the Ethics Committee on Animal Experimentation of the Federal University of Bahia (Permit Number: 34/2020 and 36/2020)

## Animals, experimental design, and diets

A total of sixty-four male Santa Inês lambs, non-castrated, with an initial age of four months and an average initial weight of 20 ± 1 kg, were used. In the first experiment (Exp1), a total of thirty-two animals were distributed in a randomized block design with four treatments. The four groups of eight animals received each one of the four experimental diets (Table 1), which were increasing concentrate levels of 400, 500, 600, and 700 g of concentrate/kg of DM. The forage contents were decreased proportionally for all treatments.

**Table 1:** Proportion of ingredients and chemical composition of the experimental diets

	Exp 1				Exp 2			
Item	Concentrate levels (g/kg DM)				Crude protein levels (g/kg DM)			
	400	500	600	700	120	140	160	180
Corn silage	600	500	400	300	500	500	500	500
Soybean meal	145	145	145	145	40	85	145	190
Ground corn	231	332	427	528	437	391	332	286
Urea/AS^1^	4	3	3	2	4	5	4	5
Mineral mix^2^	10	10	10	10	10	10	10	10
Limestone	10	10	10	10	10	10	10	10
Sodium bicarbonate	0	0	5	5	-	-	-	-
Chemical composition of the diets								
Dry matter	525	583	641	699	579	581	583	584
Organic matter	956	959	962	966	965	962	959	957
Ether Extract	27	29	30	32	31	30	29	28
Crude protein	166	165	164	163	118	141	165	188
apNDF^3^	323	289	254	220	292	290	289	286
Non-fibrous carbohydrates	451	486	522	557	451	487	522	557
Total carbohydrates	750	751	748	749	802	779	751	729
TDN^4^	680	698	712	730	738	733	728	723

^1^Urea/ammonium sulfate ratio of 9:1; ^2^Provides per kg: calcium 110 g; phosphorus 87 g; sulfur 18 g; sodium 147 g; cobalt 15 mg; copper 590 mg; chromium 20 mg; iodine 50 mg; manganese 2000 mg; molybdenum 300 mg; selenium 20 mg; zinc 3800 mg; fluorine 870 mg. ^3^Neutral detergent fiber corrected for ash and protein; TDN: total digestible nutrients estimated by the models of Cruz et al. (2021)

In the second experiment (Exp 2), another thirty-two animals were also randomly distributed among four groups of eight animals in a randomized block design. Experimental treatments were four diets formulated with increasing crude protein (CP) contents (Table 1), which were 120, 140, 160, and 180 g CP/kg DM. The diet of 160 g CP/kg DM was formulated to meet the requirements of hair sheep raised in tropical conditions, with an estimated average daily gain (ADG) of 250 g/day, in accordance with National Research Council (2007). Two other diets were formulated with CP below and one with CP above the established requirement. The diets in Exp 1 were formulated to be isonitrogenous (160 g/kg of crude protein) and to meet the nutritional requirements for lambs with an estimated potential average weight gain of 250 g/day.

Both experiments were carried out simultaneously at the same period and were derived from two performance and meat quality experiments. All animals originated from commercial farms and were kept on the experimental farm until the end of the feedlot period and their slaughter. The lambs were kept in feedlot for 57 days, preceded by a 14-days of adaptation period, during which they were adapted to the location and experimental diets. The animals were housed in individual pens (1.2 m^2^), covered with suspended and slatted floors provided with individual feeders and drinkers. Animals were fed *ad libitum* and received free access to water throughout the experimental period. During the adaptation period, the animals were identified, subjected to endo and ectoparasites control, and immunized with polyvalent vaccines against rabies and clostridial disease. Both diets used corn silage as a forage source. The concentrate was formulated using soybean meal, ground corn, urea, mineral mixture, limestone, and sodium bicarbonate (Table 1). The lambs were fed ad libitum with a total mixed ration (TMR) offered twice daily, with equal portions provided at 09:00 and 16:00 h, with refusals maintained at 10 to 15% of the feed offered (on an as-fed basis).

## Experimental procedures and sample collection

To determine the intake of each nutritional component, individual refusals were collected daily before the morning feeding and subsequently weighed on a digital scale. The feed offered, and refusals were sampled weekly during the experimental period. These samples were then stored in plastic bags, identified, and kept in a freezer at −20 °C. Thus, nutrient intake was estimated by the difference between the total amount of each nutrient in the offered feed and the amount in the refusals for DM, neutral detergent fiber corrected for ash and protein (apNDF), organic matter (OM), crude protein (CP), ether extract (EE), total carbohydrates (TC), and non-fibrous carbohydrates (NFC).

The digestibility trial was conducted on days 55 to 57 of the experimental period of each experiment, totaling three consecutive days of fecal collection, with two spot samples collected per day at 8:00, 10:00, 12:00, 14:00, 16:00, and 18:00 h (Lazzarini et al. 2016). Immediately after collection, the fecal samples were pre-dried in a forced-air oven (55 °C for 72 h) and then ground in a knife mill (Willey mill; TECNAL, São Paulo, SP, Brazil) equipped with 1 and 2-mm sieves. After grinding, composite samples were prepared proportionally based on dry weight for each animal per period, packed in identified plastic bags, and stored at room temperature.

Indigestible neutral detergent fiber (iNDF) was used as an internal marker to estimate fecal output. For this analysis, approximately 0.5 g of refusal, feces, and diet ingredient samples were weighed in triplicate and placed in non-woven textile bags (NWT; 100 g/m^2^, 50 μm; 4 × 5 cm) at a ratio of 20 mg of air-dried sample per cm^2^ of surface area. The fistulated cattle were adapted to a basal diet and bags were in situ incubated during 288 h (Reis et al. 2017). All bags were removed from the rumen, manually washed until clear water, and then partially dried in a forced-air oven (55 °C for 72 h). Subsequently, the bags were treated with a neutral detergent solution and maintained in an autoclave at 120 °C for 60 minutes, washed in boiling water and acetone. They were then pre-dried in a forced-air oven (55 °C for 72 h) and placed in a non-ventilated oven at 105 °C for 45 minutes for total drying. After, bags were destined for NDF analysis to quantify the iNDF.

Blood samples were collected on the 54th day of each experiment, four hours after the morning feeding and a few days before the end of the experimental period. After fasting, the animals were weighed at the end of the experiment on the 57th day, with the following weights: Exp1 36.54 ± 12.3 kg and Exp2 35.71 ± 6.6 kg. Approximately 10 ml of blood were collected individually in a sterile test tube with a clot activator from all animals. The samples were temporarily kept at room temperature until clot retraction occurred and then centrifuged at up to 1370 RCF for 15 minutes to obtain serum. Finally, the serum was stored at −20 °C until analysis. Spot urine samples were collected on the 54th day of each experiment approximately four hours after the morning feeding during spontaneous urination. Immediately, 10 mL of urine from each animal was filtered through cheesecloth and diluted with 40 mL of a 0.036 N sulfuric acid solution (Valadares et al. 1999). Urinary volume was estimated by analyzing creatinine concentration using a commercial assay kit (ID: 35–100; Labtest, Lagoa Santa, Minas Gerais, Brazil), with absorbance measured via spectrophotometry. Daily urinary volume was calculated assuming a creatinine excretion rate of 19.82 mg/kg BW based on values reported for crossbred Dorper × Santa Inês sheep in similar conditions and environment was this study (dos Santos et al. 2018).

## Laboratory analyses

Chemical analyses were conducted at the Animal Nutrition Laboratory of the School of Veterinary Medicine and Animal Science at the Federal University of Bahia. Samples of ingredients, refusals, and feces were analyzed according to the protocols described by the Brazilian National Institute of Science and Technology in Animal Science (INCT CA; Detmann et al. 2021). The following method numbers were used: DM (method G 003/1), ash (method M 001/2), EE (method G 005/2), and apNDF (method F 002/2).

The total nitrogen (N) content in the samples of offered ingredients, refusals, and feces was measured by the Kjeldahl method, and CP was calculated as *N* × 6.25 (INCT- CA; method *N*-001/2, Detmann et al. 2021). The OM content of forage and feeds was determined using ash values.

The NFC content of the diets was calculated according to the equation proposed by Hall (2000): NFC = 100 – [(dietary CP % - urea CP % + dietary urea %) + apNDF % + EE % + ash %]. Total carbohydrates (TC) were estimated using the equation proposed by Sniffen et al. (1992): TC = 100 - (CP % + EE % + ash %).

The iNDF provided from the NWT bags was analyzed according to the INCT CA-F 009/1 method and calculated using the equation proposed by Detmann et al. (2012).

Serum samples were analyzed for total albumin (ALB), urea, total cholesterol, triglycerides, creatinine, alkaline phosphatase (AP), alanine aminotransferase (ALT), aspartate aminotransferase (AST), and gamma-glutamyltransferase (GGT) concentrations using commercial kits (Labtest Diagnostica). Biochemical analyses were processed using a semi-automatic device (Bioplus 2000®). The device was previously calibrated with Calibra 1 in conjunction with Qualitrol 1 H universal control serum (Labtest Diagnostica). The reference values used in the study were proposed by González and Silva (2022) and the hepatic enzymes by Kaneko et al. (2008).

The glomerular filtration rate (GFR) was calculated according to Cockcroft (1976) using the formula:

  

$$\mathrm{GFR}=\mathrm{(UCr}*\mathrm{V)/PCr},$$

Where: GFR = Glomerular Filtration Rate (ml/min), Ucr = Urine creatinine concentration (mg/dL), V = Collected urine volume per minute (ml/min), PCr = Plasma or serum creatinine concentration (mg/dL).

## Statistical analysis

The data from both experiments were analyzed using the MIXED procedure of SAS (OnDemand for Academics version). The treatments applied (protein levels or concentrate levels) were considered fixed effects in the model, along with the effect of blocks. A random regression model was evaluated, considering each blood metabolite as a dependent variable and the protein or dOM intake per kg of BW as an independent variable.

In this context, the model’s slope and intercept were considered random variables to verify whether there is a linear relationship between the variables. The estimates of the intercept and slope were considered significant or not, using 0.05 as the critical probability level for type I error.

## Results

In Exp1, with the increase in concentrate in the diets, the average DM intake was 1029.2 g/day, with a standard deviation of ±152.3 g, while the average DM digestibility was 73.1%. In Exp2, with the increase in CP in the diets, the average DM intake was 1335.9 ± 204.7 g/day, with an average digestibility of 69.8%. It was observed that OM and CP intake and digestibility followed similar trends, with higher average values in Exp2 (Table 2). There was no effect of the treatments (Table 3) both in Exp1 and Exp2 on any variables (*p>*0.05), with exception for urea (mg/dL) in Exp 2, which increased as CP content increased in the diets also (*p* = 0.0002).

**Table 2:** Descriptive statistics of the intake and digestibility of diets with increasing levels of concentrate (Exp 1) or crude protein (Exp 2)

	Exp 1						Exp 2				
	Concentrate levels (g/kg DM)						Crude protein levels (g/kg DM)				
Item	Intake (g/d or g/kg BW)										
	Mean	Median	Minimum	Maximum	St-dev^2^		Mean	Median	Minimum	Maximum	St-dev^2^
Dry matter (g/d)	1029.2	1030.8	687.6	1503.8	152.3		1335.9	1337.2	930.1	1702.9	204.7
Organic matter (g/d)	992.1	987.6	663.6	1438.9	147.3		1253.8	1257.5	878.4	1596.3	192.0
Digestible organic matter (g/d)	739.4	736.6	434.9	1072.2	123.4		885.7	900.4	623.8	1136.1	161.6
dOM (g/kg BW)	20.3	20.4	13.02	31.54	2.6		44.3	43.3	28.33	70.2	7.3
Crude protein (g/d)	172.9	174.1	114.8	256.3	25.7		215.1	208.3	110.0	332.5	46.8
Crude protein (g/kg BW)	4.8	4.6	3.6	7.5	1.4		7.62	7.5	3.6	13.1	1.7
apNDF^1^ (g/d)	293.6	297.1	190.3	510.6	47.6		334.39	338.9	240.4	429.3	53.7

Digestibility (g/kg DM)											
	Mean	Median	Minimum	Maximum	St-dev^2^		Mean	Median	Minimum	Maximum	St-dev^2^
Dry matter	731.3	732.0	648.2	806.4	28.7		698.2	701.0	593.4	816.5	40.3
Organic matter	745.7	74.7	666.6	820.2	28.2		711.2	713.6	623.0	830.4	37.4
Crude protein	701.8	727.3	535.1	785.0	46.7		652.0	652.8	518.8	805.3	54.8
apNDF^1^	579.2	581.7	440.7	720.1	56.7		368.8	352.6	234.0	573.1	76.1

Neutral detergent fiber corrected for ash and protein; ^2^St-dev – standard deviation

**Table 3:** Confidence interval (α = 95%), standard-error mean (SEM) and *p*-value of the averages obtained in lambs fed with increasing levels of concentrate (Exp 1) or crude protein (Exp 2)

	Concentrate levels (g/kg DM)				Confidence interval (α = 95%)		
	400	500	600	700		SEM	*p*-value
Alkaline Phosphatase (U/L)	268.4	335.2	311.9	317.4	±58.84	28.7	0.9192
AST (U/L)	63.4	68.7	63.7	66.2	±6.71	3.2	0.9682
GGT (U/L)	65.4	67.6	64.4	73.2	±9.96	4.8	0.1210
ALT (U/L)	10.7	15.6	12.0	11.9	±3.37	1.6	0.1210
Creatinine (mg/dL)	0.70	0.68	0.79	0.84	±0.14	0.07	0.7001
Urea (mg/dL)	53.4	57.0	66.7	55.2	±8.36	4.1	0.2565
Cholesterol (mg/dL)	65.2	62.2	70.4	63.1	±6.88	3.35	0.4809
Albumin (mg/dL)	2.7	2.74	3.2	2.9	±0.23	0.11	0.4939

	Crude protein levels (g/kg DM)				Confidence interval (α = 95%)		
	120	140	160	180		SEM	*p*-value
Alkaline Phosphatase (U/L)	288.1	298.5	335.2	282.8	±41.8	20.3	0.6889
AST (U/L)	70.2	67.7	69.3	66.8	±7.28	3.54	0.5519
GGT (U/L)	65.5	61.4	67.3	57.8	±9.47	4.6	0.6257
ALT (U/L)	14.4	15	15.625	12.3	±4.51	2.2	0.4523
Creatinine (mg/dL)	0.88	0.82	0.67	0.76	±0.12	0.06	0.7628
Urea (mg/dL)	28.8	44.8	57.0	75.8	±9.88	4.8	0.0002
Cholesterol (mg/dL)	67.5	58.125	62.25	63.0	±8.15	3.9	0.5563
Albumin (mg/dL)	46.3	40.0	42.7	43.5	±31.83	0.12	0.1175

**Fig. 1:**
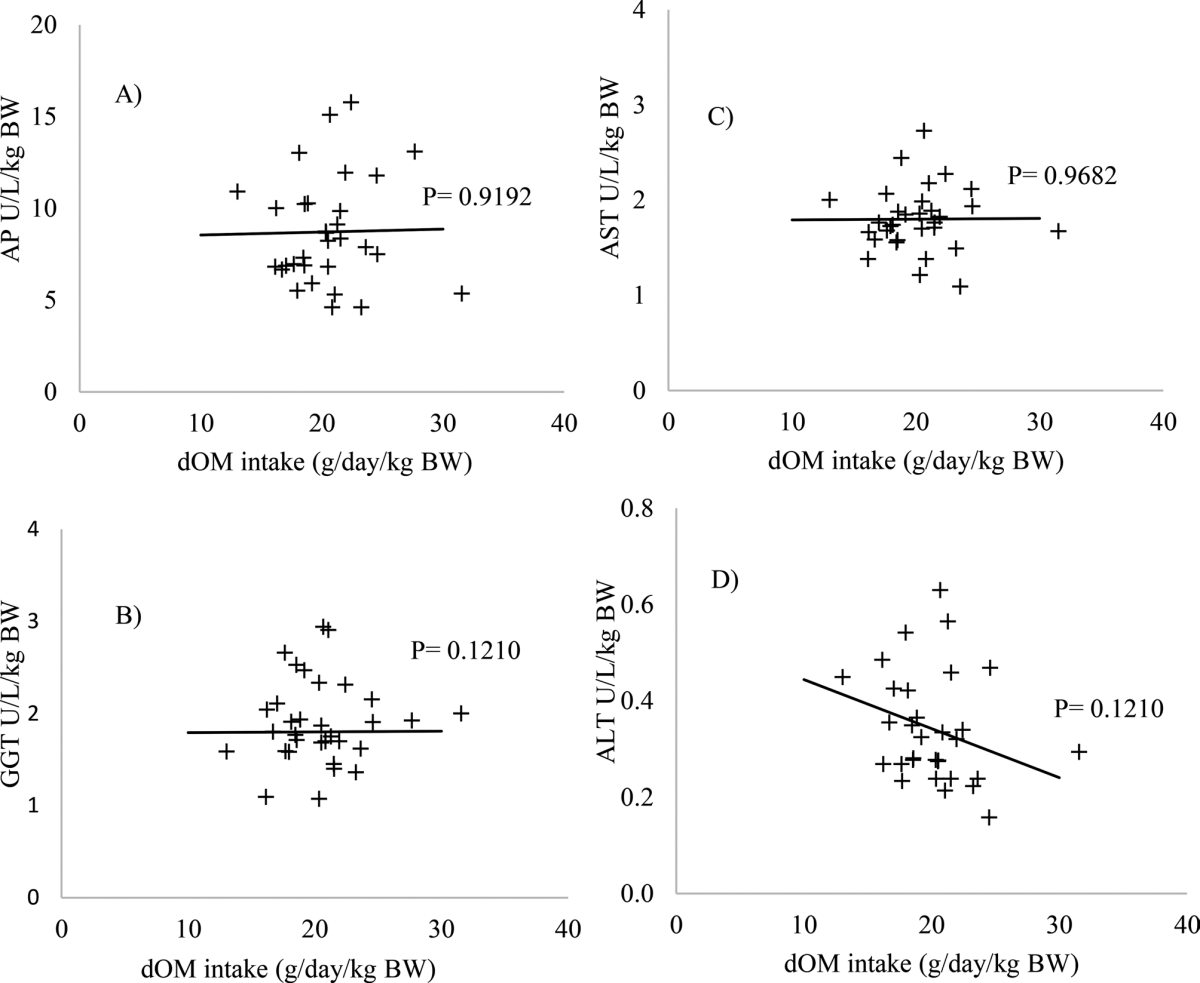
Relationship between intake of digestible organic matter (dOM) (g/day/kg BW) and the followed enzymes **A**) alkaline phosphatase (AP), **B**) gamma-glutamyl transferase (GGT), **C**) aspartate aminotransferase (AST), and **D**) alanine aminotransferase (ALT) (U/l/kg BW) in the serum of sheep

**Fig. 2:**
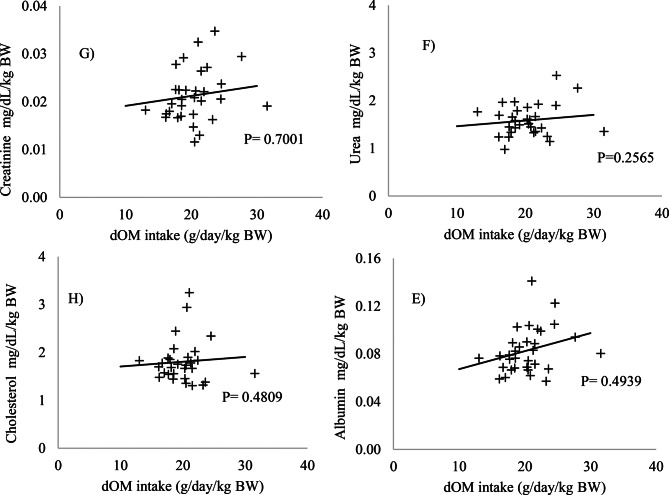
Relationship between intake of digestible organic matter (dOM) (g/day/kg BW) and the followed metabolites **E**) albumin, **F**) urea, **G**) creatinine, and **H**) cholesterol (mg/dL/kg BW) in the serum of sheep

**Fig. 3:**
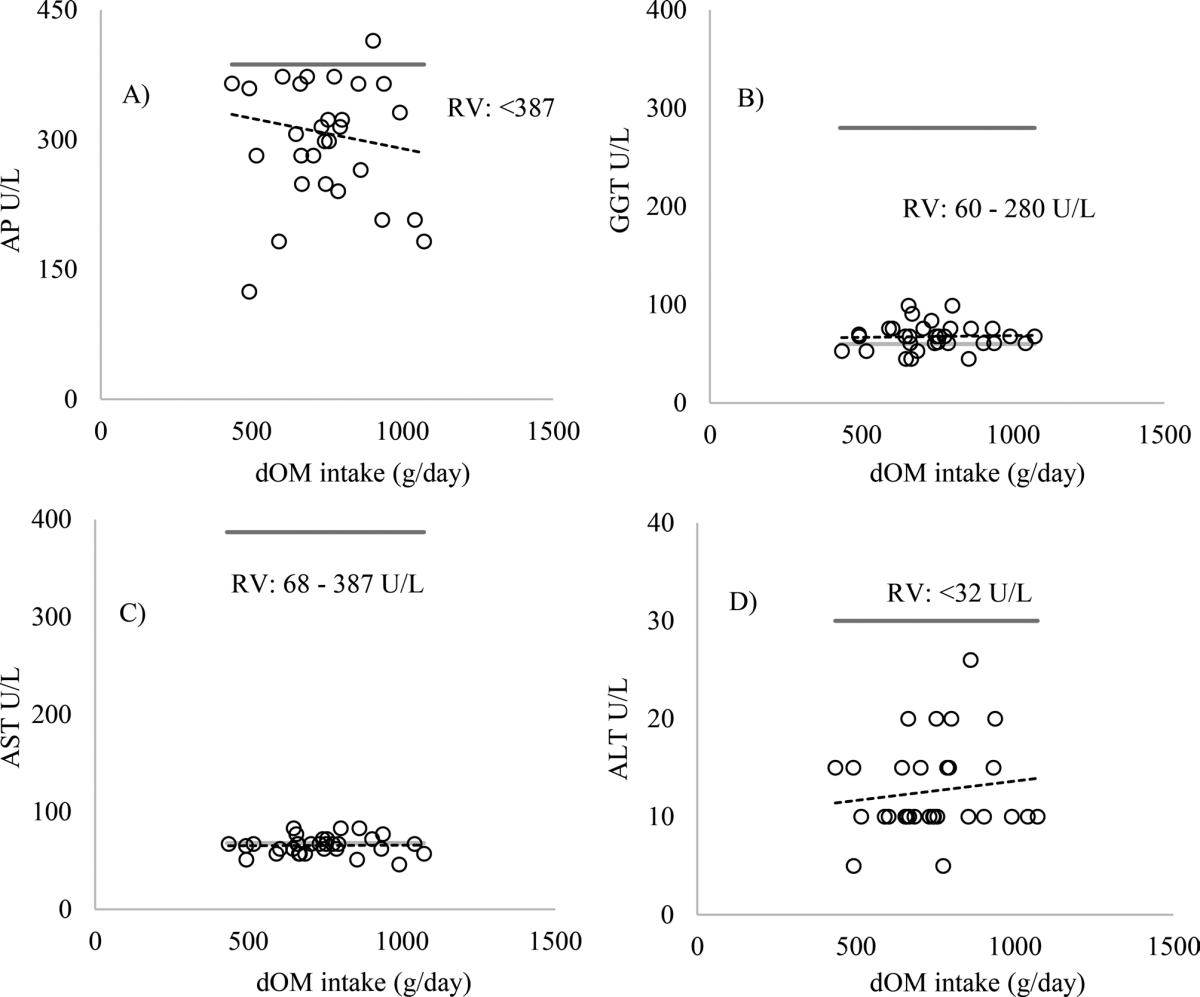
Relationship between intake of digestible organic (dOM) (g/day) and the values of enzymes **A**) alkaline phosphatase (AP), **B**) gamma-glutamyl transferase (GGT), **C**) aspartate aminotransferase (AST), and **D**) alanine aminotransferase (ALT), U/L/kg BW, in the serum of sheep. The light gray line represents the minimum reference value and the dark gray line the maximum reference value (RV): maximum (), minimum () (Kaneko et al. 2008)

**Fig. 4:**
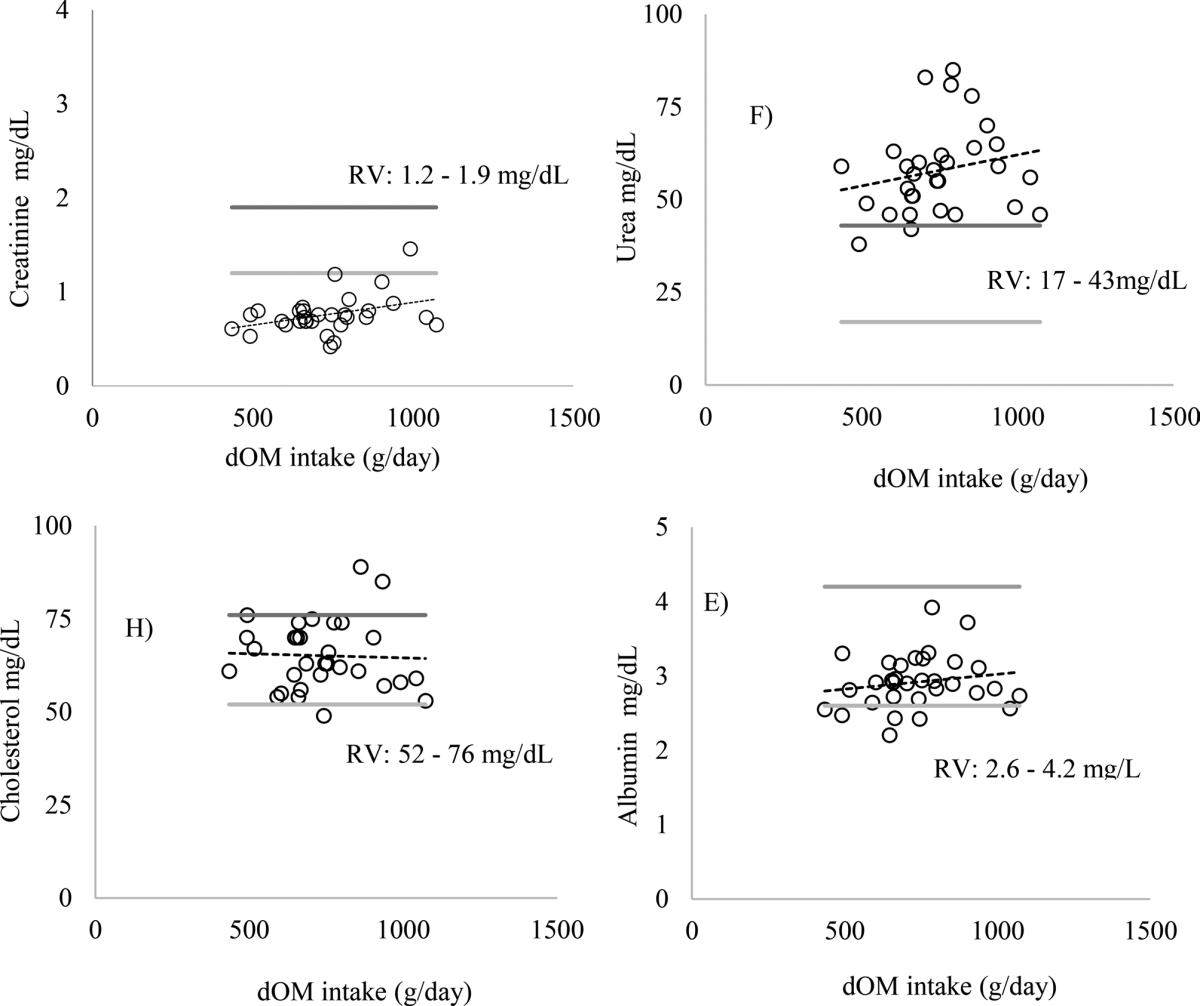
Relationship between intake of digestible organic (dOM) (g/day) and the values of the metabolites **E**) albumin, **F**) urea, **G**) creatinine, and **H**) cholesterol, mg/dL/kg BW, in the serum of sheep. The light gray line represents the minimum reference value and the dark gray line the maximum reference value (RV): maximum (), minimum () González and Silva (2022)

No significant changes were observed in the blood parameters evaluated (*p* > 0.05) (Figs. 1 and 2). The levels of the enzymes AP, AST, ALT, GGT (Fig. 3), albumin, and cholesterol (Fig. 4) remained within the ideal range, below the recommended limits for sheep, which are <387, 68–280, <32, 60–280 U/L, 2.6–4.2, and 52–76 mg/dL, respectively.

**Fig. 5:**
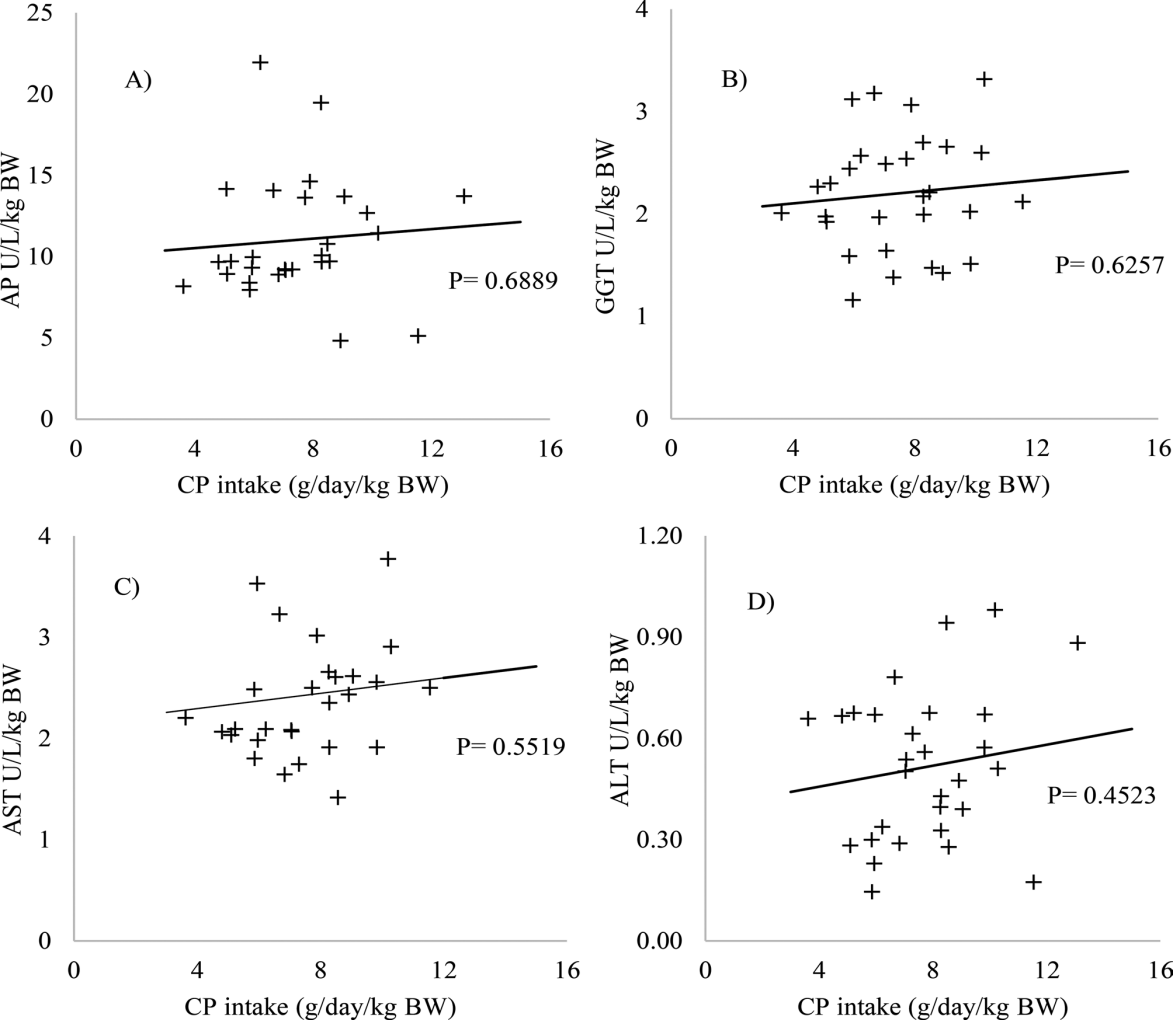
Relationship between crude protein (CP) intake (g/day/kg BW) and the followed enzymes **A**) alkaline phosphatase (AP), **B**) gamma-glutamyl transferase (GGT), **C**) aspartate aminotransferase (AST), and **D**) alanine aminotransferase (ALT), U/L/kg BW, in the serum of sheep

**Fig. 6:**
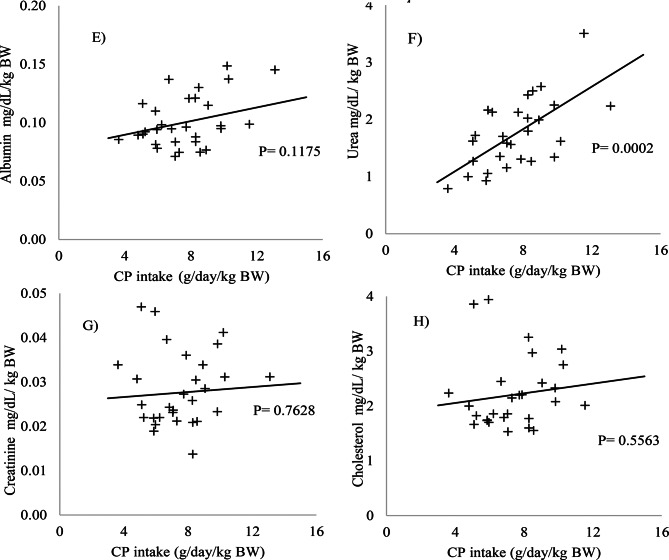
Relationship between crude protein (CP) consumption (g/day/kg BW) and the values of the metabolites **E**) albumin, **F**) urea, **G**) creatinine, and **H**) cholesterol, mg/dL/kg BW, in the serum of sheep

Urea levels remained above the ideal range (17–43 mg/dL) in Exp1 for animals that consumed a diet with an average of 16.8% CP, 884.5 g/kg BW of CP, between 434.9 and 1072.2 g/day of digestible organic matter, and an average intake of 172.9 g/day of CP. Creatinine values were below the ideal range (1.2–1.9 mg/dL), and cholesterol remained within the recommended range (52–76 mg/dL) (Fig. 4). The increase in CP intake in Exp2 promoted a significant linear increase in serum urea levels (*p* = 0.0002) (Fig. 6). However, no statistically significant differences were observed for AP, albumin, AST, ALT, GGT, creatinine, and cholesterol (Figs. 5 and 6) (*p* > 0.05).

**Fig. 7:**
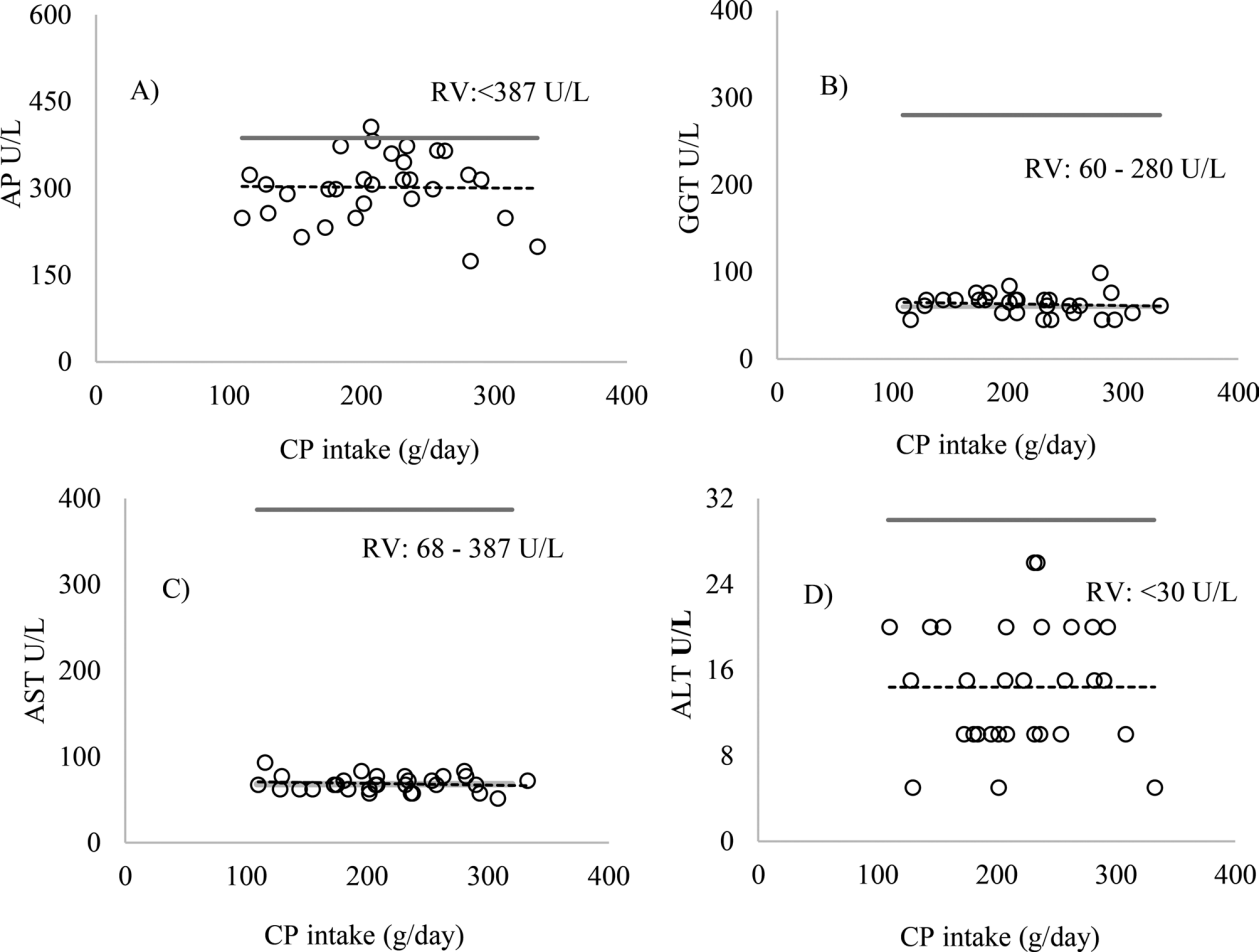
Relationship between crude protein (CP) intake (g/day) and the values of enzymes. **A**) alkaline phosphatase (AP), **B**) gamma-glutamyl transferase (GGT), **C**) aspartate aminotransferase (AST), and **D**) alanine aminotransferase (ALT), U/L/kg BW. The light gray line represents the minimum reference value and the dark gray line the maximum reference value (RV): maximum (), minimum () (Kaneko et al. 2008)

**Fig. 8:**
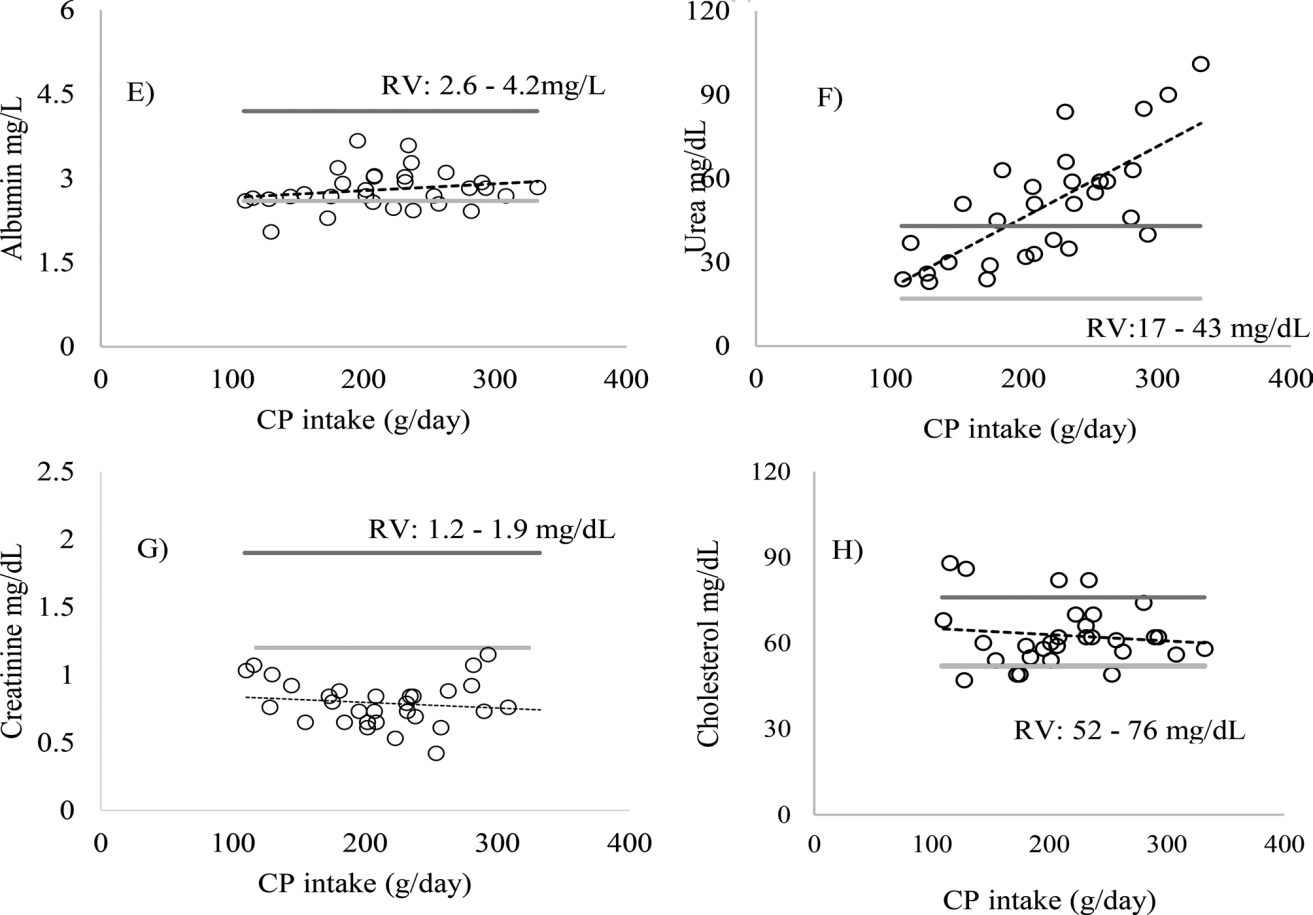
Relationship between crude protein (CP) intake (g/day) and the values of the metabolites **E**) albumin, **F**) urea, **G**) creatinine, and **H**) cholesterol in the serum of sheep. The light gray line represents the minimum reference value and the dark gray line the maximum reference value (RV): maximum (), minimum () González and Silva (2022)

**Fig. 9:**
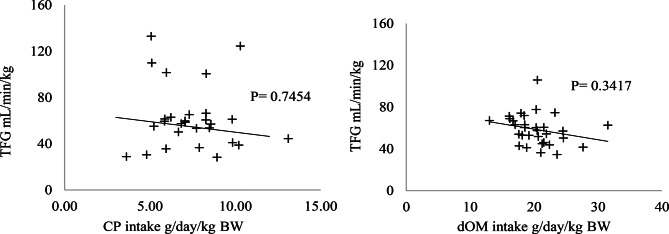
**A**) Relationship between digestible organic matter (dOM) intake (g/day/kg BW) and glomerular filtration rate (GFR) (ml/min/kg). **B**) Relationship between crude protein (CP) intake (g/day/kg BW) and glomerular filtration rate (GFR) (ml/min/kg)

In Exp2, the results were similar to Exp1, with the levels of AP, AST, ALT, GGT, albumin, and cholesterol within the ideal range (Figs. 7 and 8). Urea levels exceeded the reference values RV: 17–43 mg/dL) when CP intake was above 200 g/day, with diets ranging from 11.64% to 20.15% CP, while creatinine (RV: 1.2–1.9 mg/dL) showed values below the reference range, as shown in (Fig. 8). For the glomerular filtration rate (GFR), no changes were observed in both experiments (*p* > 0.05) (Fig. 9).

## Discussion

Blood metabolites are essential tools for verifying the adequacy of nutrient absorption, the functionality of vital organs, and the detection of any potential damage to the animal (Sá et al. 2014; Puppel and Kuczyńska 2016; Ding et al. 2016). González and Silva (2022) estimated reference ranges for blood metabolites in sheep, including cholesterol (52–76 mg/dL), creatinine (1.2–1.9 mg/dL), urea (17–43 mg/dL), and albumin (26–42 g/L). For hepatic enzymes, the recommended values are aspartate aminotransferase (AST) (68–387 U/L), alanine aminotransferase (ALT) (<30 U/L), gamma-glutamyl transferase (GGT) (60–280 U/L), and alkaline phosphatase (AP) (<387 U/L) according to Kaneko et al. (2008).

The absence of differences in serum urea levels observed in Exp1 can be attributed to the isoproteic diets. This suggests that variations in digestible organic matter intake did not significantly influence protein utilization, although blood serum urea levels were above the ideal range for all animals as recommended by González and Silva (2022) (17–43 mg/dL). When urea and creatinine levels are within the normal range, it can be inferred that renal function was not compromised by the diets offered (Sauberlich 1981; Payne and Payne 1987; Kaneko et al. 2008). However, despite elevated urea levels and low creatinine levels, the enzymes AP, AST, ALT, and GGT remained within normal ranges, suggesting the absence of liver damage, even with the increase in circulating blood urea.

Even though creatinine was not influenced by the concentrate level, the mean values were consistent above the reference value. The study of Rico et al. (1977) evaluated blood biochemical parameters in lambs aged 1.5 and 3 months and showed that young animals naturally exhibit lower serum creatinine concentrations, with mean values of approximately 0.7 mg/dL in 1.5monthold lambs and 0.9 mg/dL in 3monthold lambs, which were similar to this study. These values were lower than those typically reported in reference values for adult sheep (1.2–1.9 mg/dL), reflecting the reduced muscle mass and the stilldeveloping metabolic state characteristic of early growth. This study highlights the importance of using age specific reference ranges for lambs to avoid misinterpretation of renal function when applying values established for adult animals, but the literature is scarce is this type of work.

In Exp1, the animals consumed an average of 172.9 g/day of crude protein (CP), while the recommended intake according to the Brazilian Table of Nutritional Requirements of sheep and goats (Ovinos and 2024) for sheep in the same category as our study is 149.75 g/day of CP. Studies indicate that high-protein diets can elevate blood urea levels, suggesting an additional effort by the liver to metabolize these nutrients (Wang et al. 2020). However, in many cases, increased protein intake does not result in greater utilization of this protein by the body (Wang et al. 2021). This suggests that the increase in blood serum urea levels reflects waste and a potential overload for the liver. Nevertheless, this overload did not increase the hepatic enzymes AP, AST, ALT, and GGT, nor the creatinine levels, demonstrating that the elevated protein intake did not cause liver damage.

In Exp2, with increasing levels of CP, the animals consumed an average of 215.1 g/day, with average values of 143.0, 201.8, 248.5, and 256.47 g/day of CP when separated by treatments, an average of 1335.9 g of dry matter (DM), and a weight of 29.1 ± 11.4 kg. According to the BR-Ovinos and (2024) recommendations, uncastrated sheep with a weight of 30 kg and a daily gain of 200 g require an intake of 1125 g of DM and 149.75 g/day of CP. These values help explain why blood urea levels remained within the ideal range for animals consuming CP in quantities below their requirements. However, when CP intake exceeded the recommended levels, urea levels increased, exceeding the recommended range of 17–43 mg/dL for sheep, as suggested by González and Silva (2022).

Wang et al. (2020), evaluating increasing protein levels in the diet (125, 132, 140, 148, and 156 g/kg of CP) of confined sheep with a forage ratio of 30:70, demonstrated a significant linear increase (*p* = 0.002) in the amount of nitrogen in the form of urea present in the blood (19.27, 22.52, 22.91, 24.93, and 23.58 mg/dL).

However, the values remained within the ideal range for sheep in all treatments. When evaluating the hepatic enzymes AST and ALT to investigate liver damage, the values of all treatments were within the ideal range: AST (109.2 ± 7.2 U/L) and ALT (21.07 ± 2.0 U/L).

Enzymes such as ALT, AST, GGT, and AP are used as markers to determine liver damage, resulting in increased hepatocyte permeability (Stojević et al. 2005). González and Silva (2022) showed that the activity of these enzymes allows inferences about the location and degree of cellular damage, as their increase is directly linked to liver damage from various sources—liver, skeletal muscle, heart, and kidneys. In our study, the enzymes AP, ALT, AST, and GGT did not increase with a higher intake of dOM or CP and remained within the reference range proposed by Kaneko et al. (2008). This information helps to demonstrate that the excess circulating blood urea did not cause liver damage in confined sheep. These values are consistent with Magalhães et al. (2021), who, in a study with 80 confined lambs with a forage ratio of 50:50 containing 171 g/kg of CP, found average values of 18.6 ± 0.6, 81.45 ± 0.2, and 54.1 ± 1.3 U/L for ALT, AST, and GGT, respectively.

Cholesterol levels suggest that there were likely no deficiencies in energy metabolism and that there were no excess fat reserves being mobilized (Homem Junior et al. 2010; Rodrigues et al. 2010). Magalhães et al. (2021) found cholesterol values ranging from 67.8 to 75.2 mg/dL, which are within the acceptable range for confined lambs, according to González and Silva (2022). These values are consistent with those found in this study, both for the increased intake of dOM and CP, with respective averages of 65.09 and 62.7 mg/dL.

The glomerular filtration rate (GFR) is widely used to assess kidney function, even when not measured directly, and is commonly estimated through plasma and urinary creatinine clearance (Kamili et al. 2013). GFR can be influenced by dietary protein levels, such that a reduction in GFR may result in lower excretion of metabolites in the urine, including creatinine. To investigate potential changes in kidney function in response to diet, GFR is the most frequently employed laboratory test, serving as an important indicator of creatinine clearance (Kirsztajn 2009; Munawar et al. 2017). However, in both experiments analyzed, no significant differences in (GFR) were observed.

## Conclusion

The increase in dOM intake up to 700 g/kg DM of concentrate for lambs does not affect the liver and renal health blood markers. The high CP intake exceeding the requirements from diets up to 180 g CP/kg DM raise blood urea and reduces creatinine in feedlot growing lambs. However, under the same diets, hepatic enzymes are within the recommended ideal range, indicating the absence of liver damage.

## Data Availability

The datasets generated during and/or analyzed during the current study are available from the corresponding author on reasonable request.

## References

[CR2] Ahmed MG, AlSagheer AA, ElZarkouny SZ, Elwakeel EA (2022) Potential of selected plant extracts to control severe subacute ruminal acidosis in vitro as compared with monensin. BMC Vet Res 18(1):356. 10.1186/s12917-022-03457-4 36151574 10.1186/s12917-022-03457-4 PMC9508736

[CR1] BR–Caprinos, Ovinos (2024) Exigências nutricionais de caprinos e ovinos. Editora Scienza. 10.26626/9786556681849.2024B0001

[CR3] Cruz CH, Santos SA, de Carvalho GGP, Azevedo JAG, Detmann E, de Campos Valadares Filho S, Mariz LDS, Pereira ES, Nicory IMC, Tosto MSL, Alba HDR (2021) Estimating digestible nutrients in diets for small ruminants fed with tropical forages. Livest Sci 249:104532. 10.1016/j.livsci.2021.104532

[CR4] Detmann E, Silva LFC, Rocha GC, Palma MNN, Rodrigues JPP (2021) Métodos para análise de alimentos, 2nd edn. Suprema

[CR5] Detmann E, Souza MA, Valadares Filho SC, Queiroz AC, Berchielli TT, Saliba EOS, Cabral LS, Pina DS, Ladeira MM, Azevêdo JAG (2012) Métodos para análise de alimentos, 1st edn. Suprema

[CR6] Ding XZ, Long RJ, Zhang QM, Huang XD, Guo XS, Mi JD (2016) Blood metabolic profile in yak: a review. Afr J Biotechnol 15(2):45–52

[CR44] Dos Santos ACS, Santos SA, Carvalho GGP, Mariz LDS, Tosto M, Valadares Filho SC, Azevedo JAG (2018) A comparative study on the excretion of urinary metabolites in goats and sheep to evaluate spot sampling applied to protein nutrition trials. J Anim Sci 96:3381–3397. 10.1093/jas/sky198 10.1093/jas/sky198 PMC6095378 29767729

[CR9] González FHD, Silva SC (2022) Introdução à bioquímica clínica veterinária, 4th edn. Editora da UFRGS

[CR10] Homem Junior AC, Ezequiel JMB, Fávaro VR, Oliveira PSN, D’Aurea AP, Santos VC, Gonçalves JS (2010) Fermentação ruminal de ovinos alimentados com alto concentrado e grãos de girassol ou gordura protegida. Arq Bras Med Vet Zootec 62(1):144–153. 10.1590/S0102-09352010000100020

[CR11] Kamili A, Bengoumi M, Oukessou M, Lefebvre B (2013) Assessment of glomerular filtration rate in normally hydrated anddehydrated dromedary camel by plasma exogenous creatinine clearance test. Emir J Food Agric 25(4):295–303. 10.9755/ejfa.v25i4.15501

[CR12] Kaneko JJ, Harvey JW, Bruss ML (Eds.) (2008) Clinical biochemistry of domestic animals, 6th edn. Academic

[CR13] Katz NR (1992) Metabolic heterogeneity of hepatocytes across the liver acinus. J Nutr 122(Suppl. 3):843–849. 10.1093/jn/122.suppl_3.843 1542056 10.1093/jn/122.suppl_3.843

[CR14] Kirsztajn GM (2009) Avaliação de função renal. J Bras Nefrol 31(Suppl. 1):14–17

[CR43] Lazzarini I, Detmann E, Sampaio CB, Paulino MF, Valadares Filho SDC, Souza MA, Queiroz AC (2016) Intake and digestibility in cattle fed low-quality tropical forage and supplemented with nitrogenous compounds. Trop Anim Health Prod 48:1231–1238. 10.1007/s11250-016-1075-1 10.1007/s11250-010-9581-7 20414721

[CR16] Magalhães TS, Carvalho GGP, Santos EM, Lima AES, Freitas Junior JE, Pina DS, Santos SA, Pinto LFB, Mourão GB, Soares FDS, Pereira TCDJ, Leite LC (2021) Health concerns of lambs fed cottonseed hulls combined with chitosan by examining the blood metabolic profile and histopathology of the kidney, liver, and rumen. Vet Med 66(11):470–480. 10.17221/194/2020-VETMED 10.17221/194/2020-VETMED PMC12178654 40546811

[CR17] Meijer AJ, Lamers WH, Chamuleau RA (1990) Nitrogen metabolism and ornithine cycle function. Physiol Rev 70(3):701–748. 10.1152/physrev.1990.70.3.701 2194222 10.1152/physrev.1990.70.3.701

[CR19] Munawar SH, Iqbal Z, Manzoor Z (2017) Determination of renal handling of marbofloxacin in Lohi sheep (*Ovis aries*) following a single intravenous administration. Iran J Vet Res 18(1):49–55 28588633 PMC5454579

[CR20] National Research Council (2007) Nutrient requirements of small ruminants: sheep, goats, cervids, and New world camelids. National Academies Press. 10.17226/11654

[CR21] Onasanya GO, Oke FO, Sanni TM, Muhammad AI (2015) Parameters influencing haematological, serum and bio-chemical references in livestock animals under different management systems. OJVM 5(8):181–189. 10.4236/ojvm.2015.58025

[CR22] Payne JM, Payne S (1987) The metabolic profile test. Oxford University Press

[CR23] Puppel K, Kuczyńska B (2016) Metabolic profiles of cow’s blood; a review. J Sci Food Agric 96(13):4321–4328. 10.1002/jsfa.7779 27129620 10.1002/jsfa.7779

[CR25] Reis MJ, Santos SA, Prates LL, Detmann E, Carvalho GGP, Santos ACS, Rufino LM, Mariz LD, Neri F, Costa E (2017) Comparing sheep and cattle to quantify internal markers in tropical feeds using in situ ruminal incubation. Anim Feed Sci Technol 232:139–147. 10.1016/j.anifeedsci.2017.08.013

[CR26] Rico AG, Braun JP, Bénard P (1977) Blood reference values in the lamb. Reproduction nutr Développement 17(2):327–336

[CR27] Rodrigues MRC, Rondina D, Araújo ADA, Arruda IJ, Silva LM, Nunes-Pinheiro DCS, Fernandes AAO (2010) Metabolic responses and sex hormone levels in lambs fed dehydrated cashew apple pomace. Ciência Anim 20(1):1–10

[CR28] Russell AJF (1991) Body condition scoring of sheep. In: Boden E (ed) *Sheep and goat practice*. Baillière Tindall. pp 3–6

[CR31] Sauberlich HE (1981) Laboratory procedures used in vitamin nutritional assessment. Nutritional Assess 65–85

[CR32] Schultz EB, Macedo Júnior GDL, Oliveira KA, Siqueira MTS, Conceição AR, Sousa LF (2022) Intervalo de referência de parâmetros bioquímicos de ovelhas lactantes nos trópicos. Semina Ciênc Agrár 43(6):2415–2424. 10.5433/1679-0359.2022v43n6p2415

[CR29] Sá JL, Barrêto Júnior RA, Ferreira ACH, Campos ACN (2014) Blood metabolic profile of ruminants: a review. Revista Brasileira de Saúde e Produção Anim 15(2):433–445

[CR33] Sniffen CJ, O’Connor JD, Van Soest PJ, Fox DG, Russell JB (1992) A net carbohydrate and protein system for evaluating cattle diets: II. Carbohydrate and protein availability. J Anim Sci 70(11):3562–3577. 10.2527/1992.70113562x 1459919 10.2527/1992.70113562x

[CR34] Stojević Z, Piršljin J, Milinković-Tur S, Zdelar-Tuk M, Beer Ljubić B (2005) Activities of AST, ALT and GGT in dairy cows. Vet Arh 75(1):67–73

[CR36] Valadares RFD, Broderick GA, Valadares Filho SC, Clayton MK (1999) Effect of replacing alfalfa silage with high moisture corn on ruminal protein synthesis estimated from excretion of total purine derivatives. J Dairy Sci 82(12):2686–2696. 10.3168/jds.S0022-0302(99)75525-6 10629816 10.3168/jds.s0022-0302(99)75525-6

[CR39] Wang Y, Wang Q, Dai C, Li J, Huang P, Li Y, Ding X, Huang J, Hussain T, Yang H (2021) Effect of dietary protein level on growth, carcass characteristics, serum biochemical index, and meat quality of hu male lambs. Small Ruminant Res 194:106294. 10.1016/j.smallrumres.2020.106294

[CR45] Wang Y Zhang X Li F Li C Wang Z (2020) Effects of dietary protein levels on nitrogen utilization and rumen fermentation in sheep. *Small Rumin Res* 192:106208. 10.1016/j.smallrumres.2020.106208

[CR42] Xue B, Wang Z, Li J (2025) Systemic immune‑inflammation index as a prognostic marker for chronic Hepatitis B with non‑alcoholic fatty liver disease. J Infect Dev Ctries 19:315–324. 10.3855/jidc.18676 10.3855/jidc.19636 40063759

[CR41] Zhang X, Wu X, Chen W, Zhang Y, Jiang Y, Meng Q, Zhou Z (2017) Growth performance and development of internal organ, and gastrointestinal tract of calf supplementation with calcium propionate at various stages of growth period. PLoS One 12(7):e0179940. 10.1371/journal.pone.0179940 10.1371/journal.pone.0179940 PMC5503182 28692656

